# SMO expression level correlates with overall survival in patients with malignant pleural mesothelioma

**DOI:** 10.1186/1756-9966-32-7

**Published:** 2013-02-05

**Authors:** Yi Zhang, Jianxing He, Fang Zhang, Hui Li, Dongsheng Yue, Changli Wang, David M Jablons, Biao He, Natalie Lui

**Affiliations:** 1 Department of Thoracic Surgery, Xuanwu Hospital, Capital Medical University, 100053, Beijing, China; 2 Department of Cardiothoracic Surgery, The First Affiliated Hospital of Guangzhou Medical College, State Key Laboratory of Respiratory Disease, Guangzhou, China; 3 Zhejiang Provincial Key Laboratory of Applied Enzymology, Yangtze Delta Region Institute of Tsinghua University, 314006, Jiaxing, Zhejiang, China; 4 Thoracic Oncology Program, Department of Surgery, Helen Diller Family Comprehen-sive Cancer Center, University of California, 94143, San Francisco, CA, USA; 5 Department of Lung Cancer, Tianjin Medical University Cancer Institute & Hospital, 300060, Tianjin, China

**Keywords:** Mesothelioma, Hedgehog signaling, Prognosis, Proliferation suppression

## Abstract

**Background:**

Malignant mesothelioma is an aggressive, treatment-resistant tumor arising from mesothelium of pleura, peritoneum and pericardium. Despite current combined regimen, its prognosis remains dismal, calling for more effective targeted therapies. We investigated whether aberrant Hh activation may play a role in mesothelioma.

**Methods:**

SMO and SHH expression levels were analyzed in 46 mesothelioma tissue specimens with real-time RT-PCR, and correlation with survival was analyzed with univariate and multivariate Cox proportional hazards models, Kaplan-Meier survival curves, and the log-rank test. We also examined multiple mesothelioma cell lines for SMO expression and the effect of Hh inhibition by a specific SMO antagonist on cell proliferation by MTS assay.

**Results:**

We observed strong correlation between higher SMO and SHH expression levels with poorer overall survival. Remarkably, Hh inhibition by a specific SMO inhibitor significantly suppressed cell proliferation in the mesothelioma cell lines examined.

**Conclusion:**

Our data strongly support that Hh signaling deregulation plays critical roles in proliferation of mesothelioma, and consistently exerts significant impact on prognosis of the disease. Therefore our findings revealed the hitherto unappreciated role of Hh activation in mesothelioma, and pinpointed Hh signaling antagonist as a potential new therapy against this devastating disease.

## Background

Malignant mesothelioma is an aggressive, treatment-resistant tumor, arising from transformed mesothelial cells lining the pleura, peritoneum and pericardium. Athough relatively a rare disease, its incidence rate is increasing throughout the world [1,2]. Its major risk factor is asbestos exposure, besides it can also be caused by ionizing radiation, erionite exposure, chest injuries, and presumably SV40 virus [3]. Patients with malignant pleural mesothelioma (MPM) usually present with shortness of breath and chest pain with pleural effusions. Patients are diagnosed with cytopathology of mesothelioma effusions or fine-needle aspirations, and histopathology is often required to establish the diagnosis [4]. Despite the current regimen of surgical resection, chemotherapy, and radiation therapy for treating MPM, the prognosis remains dismal, with median survival being 9–12 months from diagnosis [3]. Therefore developing new molecular targeted therapies may pose promise for this devastating illness.

The pathogenic mechanisms underlying mesothelioma involve deregulation of multiple signaling pathways, including activation of multiple receptor tyrosine kinases such as the epidermal growth factor receptor (EGFR) family and MET, and subsequent deregulations of mitogen-activated protein kinase (MAPK) and phosphatidylinositol-3-kinase (PI3K)-AKT signaling cascades, the TNF-α / NF-κB survival pathway, Wnt signaling, and loss of tumor suppressors such as Neurofibromatosis type 2(NF2), p16^INK4A^, and p14^ARF^[5]–[7]. Understanding mechanisms of the dysregulated signaling pathways allows strategies for development of targeted new therapies against this devastating disease.

It has been recently reported that sonic hedgehog (Hh) signaling, another important pathway during development and tumorigenesis, is aberrantly activated in MPM, and inhibition of hedgehog signaling suppresses tumor growth [8]. Deregulated Hedgehog (Hh) pathway activation has been implicated in several human cancers including glioma, basal cell carcinoma, medulloblastoma, lung, breast, pancreatic and gastric cancers [9]–[14]. The Hh family of proteins controls multiple fundamental cellular functions, including cell proliferation and survival, body patterning and organ morphogenesis during embryonic development [9], [13]–[16]. Hh signaling is orchestrated by two trans-membrane receptors, Patched (Ptch1) and Smoothened (SMO). In the absence of the Hh ligand, PTCH1 inhibits SMO, causing cleavage of GLI1 to the N-terminal repressor form. Once Hh binds to PTCH1, the inhibitory effect on SMO is released, causing active full-length GLI1 to transport into the nucleus and activate transcription of the Hh target genes in a context- and cell-type specific manner, including GLI1, PTCH1, HHIP and C-MYC [13]–[16]. Targeted inhibition of aberrant Hh signaling leads to suppression of cancer stem cells awakened and propelled by inappropriate Hh signaling [10,11,16].

We propose that the Hh signaling pathway may play an essential role during pathogenesis of MPM. To test this hypothesis, we measured SMO and SHH expression levels in MPM tissue specimens, and studied the relation of those expression levels with regard to overall survival. We also examined multiple mesothelioma cell lines for SMO expression and their cell proliferation responses to a specific SMO inhibitor. We therefore aim to better elucidate the role of Hh signaling in the tumorigenesis of MPM, and such finding may lead to development of improved molecular targeted therapies against this fatal disease.

## Methods

### Patients

We identified patients who underwent surgical resection for malignant pleural mesothelioma at our institution from April 2000 to January 2010 and had a tissue specimen available in our tissue bank. Clinical and histological data were obtained by review of electronic medical records and entered prospectively into our tissue bank database. Vital status was obtained through the Social Security Death Master File. Overall survival was calculated from the date of surgery. Our institutional review board approved this study.

### RNA extraction and real-time RT-PCR

Total RNA was isolated from MPM tissue samples using the RNeasy kit (Qiagen). Genomic DNA contamination was eliminated by DNase I treatment. 250 ng of RNA was reverse transcribed using the iScript cDNA synthesis kit (Bio-Rad). The resulting cDNAs were analyzed with real-time RT-PCR using Gene Expression Assays in a 7900 Real-Time PCR System (Applied Biosystems) for 40 cycles (96°C for 15 seconds and 60°C for 1 minute). Gene expressions were normalized to 18S rRNA expression.

### Immunohistochemistry (IHC)

Peroxidase-based immunohistochemistry using paraffin-sections was performed per standard protocol. Smo antibody (abcam, ab72130) and Shh antibody (abcam, ab19897) were employed following the manufacturer's instructions. These slides were then mounted in Citifluor.

### Cell lines and cell culture

Mesothelioma cell lines NCI-H28, REN, and H290 were cultured in RPMI 1640 (Life Technology, Carlsbad, CA) supplemented with 10% FBS and penicillin (100 IU/ml) and streptomycin (100 ug/ml), at 37°C in a humid incubator with 5% CO2. Cells were seeded one day before treatment with cyclopamine (Selleckchem) at 10 uM and 20 uM or vehicle (DMSO) for 72 hours. Cells were subjected to proliferation assays at 0, 24, 48 and 72 hours after drug treatment.

### Cell proliferation assay

Cells will be treated with Cyclopamine at indicated doses in 96-well plates for 6–7 days. Cell proliferation was assayed by MTS assay (Promega) according to the manufacturer’s protocol and as described previously [17]. The quantity of formazan product as measured by the absorbance at 490 nm is directly proportional to the number of living cells in culture. Data are representative of at least 3 independent experiments with similar results.

### Western blotting

Whole cell lysates were resolved by SDS-PAGE and transferred to nitrocellulose membranes for immunoblotting with the indicated antibodies: α-human SMO mouse monoclonal antibody (Sigma), α-ß-actin mouse monoclonal antibody (Sigma) as described previously [18]. Data represent three independent experiments with consistent results.

### Survival and statistical analyses

Survival analysis was performed using univariate and multivariate Cox proportional hazards models, Kaplan-Meier survival curves, and the log-rank test. For the Cox proportional hazards models, age and sex were included in the multivariate model a priori. Race, histological type, stage, smoking status were included in the multivariate model only if the p-value was less than 0.10 in the univariate analysis. For all statistical tests, a two-sided alpha level less than 0.05 was considered statistically significant. Analyses were performed using Stata version 11.

## Results and discussion

### Patients

Forty-six patients underwent surgical resection for malignant pleural mesothelioma at our institution, had tissue specimens deposited at our tissue bank and available for use. Patient baseline characteristics were summarized as in Table 1.

**Table 1:** Patient baseline characteristics

	**All patients (N = 46)**
**Age, mean ± SD—yr.**	**67.2 ± 10.7**
**Sex—no. (%)**	
**Female**	**11 (24)**
**Male**	**35 (76)**
**Race—no. (%)**	
**White**	**36 (78)**
**Non-white**	**10 (22)**
**Smoking status—no. (%)**	
**Never**	**13 (28)**
**Ever**	**27 (59)**
**Missing**	**6 (13)**
**Histologic type—no. (%)**	
**Epithelioid**	**39 (85)**
**Sarcomatous**	**2 (4)**
**Other**	**5 (11)**
**Stage—no. (%)**	
**I**	**5 (11)**
**II**	**8 (17)**
**III**	**11 (24)**
**IV**	**3 (7)**
**Missing**	**19 (41)**

### SMO and SHH expression analysis

**Figure 1:**
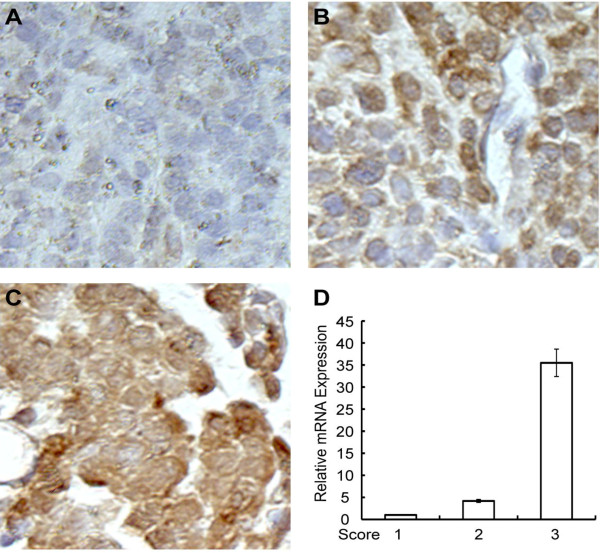
**IHC analysis of Smo protein expression in mesothelioma tissue samples. A**-**C**: Representative images of IHC for evaluating Smo protein expression level with score of 1,2 and 3. **A**, 1-low level; **B**, 2-intermediate level; **C**, 3-high level. **D**, RT-PCR measuring Smo mRNA expression level of corresponding samples of 1–3 as in **A**-**C**.

SMO and SHH expression levels were evaluated at both mRNA and protein expression levels. Protein expression levels examined by Immunohistochemistry (IHC) correlated well with mRNA levels assessed by RT-PCR (examples are shown in Figure 1). SMO expression level was determined for all 46 patients, whereas SHH expression level was determined for 23 patients. Since SMO and SHH expression level encompassed such a wide range, we chose the median level from the tumor samples as a good initial threshold to investigate the importance of SMO and SHH. Separated apart by the median level, 23 (50%) samples above the median were named as the "high" category, while 23 (50%) samples below the median were named as the "low" category. The number of samples in each category is displayed in the risk table below each Kaplan-Meier survival curve.

### Survival analysis

**Figure 2:**
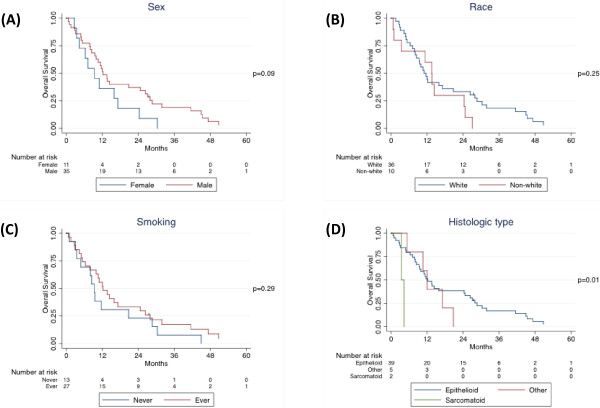
Kaplan-Meier survival curves by (A) sex, (B) race, (C) smoking status, and (D) histological type.

**Figure 3:**
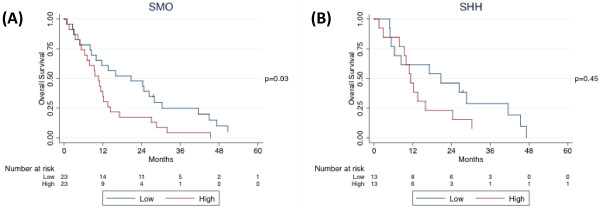
Kaplan-Meier survival curves by (A) SMO and (B) SHH expression levels.

Median follow-up time was 11.8 months (inter-quartile range, 6.3 to 27.0 months). Forty-five patients died, including 31 patients who died within two years of their operations. In the univariate Cox proportional hazards model, sex and histological type were significantly associated with overall survival, and these variables were included in the multivariate model (Table 2). Age was not significantly associated with overall survival, however, this variable was included in the multivariate model a priori. Race, smoking status, and stage were not significantly associated with overall survival, and these variables were not included in the multivariate model. In the univariate model, higher SMO expression levels were associated with worse overall survival (p = 0.05). Kaplan-Meier survival estimates confirmed these results (Figures 2 and 3A).

**Table 2:** Univariate and multivariate Cox proportional hazards model

	**Univariate analysis**			**Multivariate analysis**		
	**Hazard ratio**	**95% CI**	**p-value**	**Hazard ratio**	**95% CI**	**p-value**
**Age (10 years)**	**0.84**	**0.61-1.16**	**0.28**	**0.82**	**0.57-1.17**	**0.28**
**Sex**						
**Female**	**1**			**1**		
**Male**	**0.55**	**0.27-1.12**	**0.10**	**0.75**	**0.33-1.74**	**0.50**
**Histologic type**						
**Epithelioid**	**1**		**0.04**	**1**		**0.08**
**Sarcomatous**	**7.76**	**1.54-39.0**	**0.01**	**7.26**	**1.25-42.1**	**0.03**
**Other**	**1.53**	**0.58-4.00**	**0.39**	**1.38**	**0.52-3.69**	**0.52**
**SMO expression level**	**1.05**	**1.00-1.10**	**0.05**	**1.06**	**1.00-1.12**	**0.03**

In the multivariate Cox proportional hazards model, SMO expression level remained associated with worse survival (Table 2). However, sex was no longer associated with overall survival (p = 0.50) and histological type was less strongly associated with overall survival (p = 0.08). After adjusting for age, sex, and histological type, the hazard ratio and significance of SMO expression level increased compared to the univariate model (p = 0.03).

SHH expression level was analyzed separately because data was only available for 26 patients. In the univariate model, SHH expression level was significantly associated with overall survival. Increase in SHH expression level strongly correlates with elevated risk of death (95% CI, 1-28%; p = 0.04; data not shown). When SHH expression level was dichotomized at the median, log-rank test was not significant (p = 0.45), although the Kaplan-Meier survival curve showed separation after 12 months (Figure 3B).

After including SHH expression level in the multivariate model above, SHH expression level remained significant and even increased the significance of SMO expression level. After adjusting for age, sex, and histological type, increase in SMO expression level strongly correlates with increase in risk of death (95% CI, 8-72%; p = 0.009; data not shown); and so does increase in SHH expression level (95% CI, 1-26%; p = 0.04; data not shown). Histological type was no longer associated with overall survival (p = 0.87).

### SMO Inhibition suppresses mesothelioma cell proliferation

**Figure 4:**
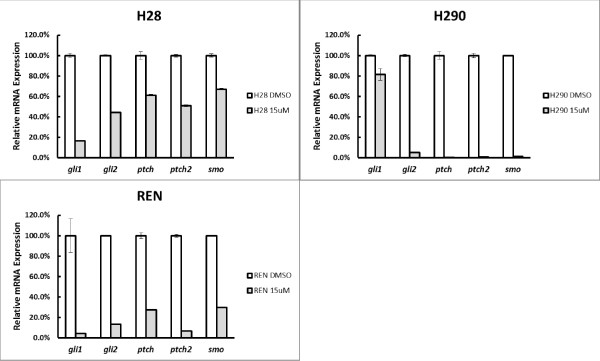
**Quantitative RT-PCR analysis of Shh pathway effectors in mesothelioma cell lines treated with cyclopamine.** Cells were treated with 15 uM cyclopamine for 72 hrs. RNA was then collected for cDNA synthesis and quantitative PCR. Actin was used as an internal control for normalization.

To assess the role of Hh signaling in tumor growth of mesothelioma, we utilized a small molecule Hh signaling inhibitor cyclopamine which specifically antagonizes SMO receptor [11]. Three mesothelioma cell lines were treated with cyclopamine and examined for expression of several key effectors of the SHH pathway. Expression of all Gli downstream effector genes (including GLI1, GLI2, PTCH, PTCH2) was suppressed, suggesting the specificity of cyclopamine in inhibiting the SHH pathway (Figure 4).

**Figure 5:**
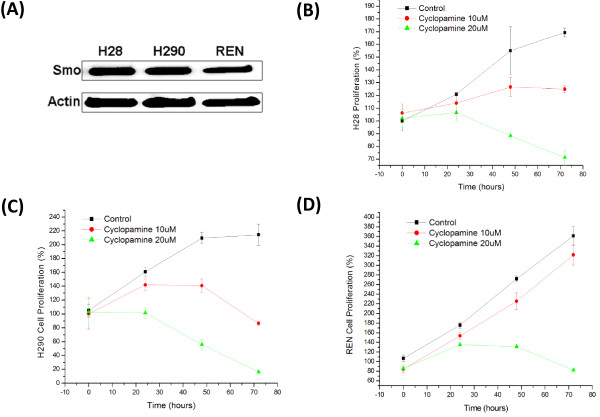
**Analysis of SMO expression and function in mesothelioma cell lines. (A)** Western analysis of SMO expression in mesothelioma cell lines. **(B-D)** MTS proliferation assay of mesothelioma cell lines following SMO inhibitor cyclopamine treatment.

We observed relatively high level of endogenous SMO expression in all three mesothelioma cell lines examined, including H28, H290 and REN (Figure 5A). Notably, Cyclopamine treatment significantly suppressed proliferation of these mesothelioma cells in a dose-dependent manner (Figure 5B-D). These results strongly support that Hh signaling plays essential role in mesothelioma cell proliferation.

### Role of Hh activation in mesothelioma

**Figure 6:**
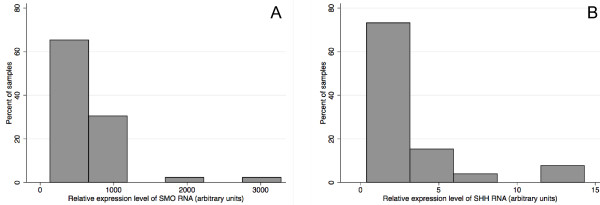
**Real-time RT-PCR analysis of expression level of (A) SMO and (B) SHH in MPM tissue samples.** X-axis represents Relative expression level of SMO (**A**) or SHH (**B**) mRNA (arbitrary units). Y-axis represents percentage of the MPM tissue samples analyzed.

Hh signaling plays pivotal roles in development and in cancer. It is implicated in tumorigenesis of multiple human cancers. However, whether Hh signaling plays essential roles in mesothelioma remains elusive. We have analyzed both mRNA and protein expression profiles of mesothelioma tumor samples from 46 patients, and showed that SHH and SMO expression was spreading over a wide range of expression levels (Figure 6). To assess whether Hh signaling activation may impact on the prognosis of mesothelioma patients, we carried out univariant and multivariant COX proportional hazard ratio analysis. Interestingly, we observed that higher SMO expression levels are strongly associated with worse overall survival in malignant pleural mesothelioma after adjusting for age, sex, and histological type (Figures 2, 3A). Consistently, higher SHH expression level correlates with worse survival in a smaller number of patients (Figure 3B). Although our results are limited by relatively small number of patients, due to the relatively low incidence of MPM, our data strongly support that Hh signaling plays indispensable roles in mesothelioma, and exerts significant impact on the prognosis of mesothelioma patients.

As deregulated Hh signaling pathway has been implicated in many different types of cancer, and inhibition of Hh signaling leads to suppression of tumor growth [10,11], we addressed whether Hh signaling plays critical roles in proliferation of mesothelioma cells. Remarkably, we observed elevated endogenous SMO expression in 3 mesothelioma cell lines tested (Figure 5A). Furthermore, utilizing a specific Hh inhibitor cycloplamine, which significantly suppressed expression of Gli downstream targets (Figure 4), we observed significant inhibition of cell proliferation in all 3 mesothelioma cell lines examined (Figure 5B-D). These data indicate that aberrant Hh activation plays critical roles in tumor cell proliferation in mesothelioma, consistent with recent data by Shi Y et al. [8].

## Conclusions

Taken together, our results demonstrated a strong association between higher SMO and SHH expression levels with poorer overall survival. Furthermore, we showed inhibition of Hh signaling blocked cell proliferation in multiple mesothelioma cell lines, strongly supporting that aberrant Hh signaling is essential for tumor growth in mesothelioma. Therefore our findings revealed the hitherto unappreciated roles of Hh activation in MPM, and pinpointed Hh signaling antagonist as a potential new therapy against this devastating disease.

## Competing interests

All authors have no competing financial interests.

## Authors’ contributions

YZ carried out the statistic analysis and drafting of the manuscript. JH carried out the cell cultures and cell proliferation assays, Western blotting and drafting of the manuscript. FZ carried out the RNA extractions and Real-time RT-PCR assays, drafting and revising the manuscript. HL participated in the statistic analysis. DMJ conceived of the study and supervised the projects. BH designed the experimental approaches and coordinated the project progression. NL participated in the cell proliferation assay and the Western Blot assay. All authors read and approved the final manuscript.
